# Towards Reliable and Energy-Efficient Incremental Cooperative Communication for Wireless Body Area Networks

**DOI:** 10.3390/s16030284

**Published:** 2016-02-24

**Authors:** Sidrah Yousaf, Nadeem Javaid, Umar Qasim, Nabil Alrajeh, Zahoor Ali Khan, Mansoor Ahmed

**Affiliations:** 1 COMSATS Institute of Information Technology, Islamabad 44000, Pakistan; sidrahyousafuetpeshawar@yahoo.com (S.Y.); mansoor@comsats.edu.pk (M.A.); 2 Cameron Library, University of Alberta, Edmonton, AB T6G 2J8, Canada; umar.qasim@ualberta.ca; 3 College of Applied Medical Sciences, King Saud University, Riyadh 11633, Saudi Arabia; nabil@ksu.edu.sa; 4 Faculty of Engineering, Dalhousie University, Halifax, NS B3J 4R2, Canada; Zahoor.Khan@dal.ca; 5 Computer Information Science, Higher Colleges of Technology, Fujairah 4114, United Arab Emirates

**Keywords:** wireless body area networks, energy consumption, reliability, relaying, incremental cooperative communication, routing

## Abstract

In this study, we analyse incremental cooperative communication for wireless body area networks (WBANs) with different numbers of relays. Energy efficiency (EE) and the packet error rate (PER) are investigated for different schemes. We propose a new cooperative communication scheme with three-stage relaying and compare it to existing schemes. Our proposed scheme provides reliable communication with less PER at the cost of surplus energy consumption. Analytical expressions for the EE of the proposed three-stage cooperative communication scheme are also derived, taking into account the effect of PER. Later on, the proposed three-stage incremental cooperation is implemented in a network layer protocol; enhanced incremental cooperative critical data transmission in emergencies for static WBANs (EInCo-CEStat). Extensive simulations are conducted to validate the proposed scheme. Results of incremental relay-based cooperative communication protocols are compared to two existing cooperative routing protocols: cooperative critical data transmission in emergencies for static WBANs (Co-CEStat) and InCo-CEStat. It is observed from the simulation results that incremental relay-based cooperation is more energy efficient than the existing conventional cooperation protocol, Co-CEStat. The results also reveal that EInCo-CEStat proves to be more reliable with less PER and higher throughput than both of the counterpart protocols. However, InCo-CEStat has less throughput with a greater stability period and network lifetime. Due to the availability of more redundant links, EInCo-CEStat achieves a reduced packet drop rate at the cost of increased energy consumption.

## 1. Introduction

Wireless body area networks (WBAN) are one of the emerging technologies that has the prospect to significantly enhance healthcare monitoring and related medical procedures. WBANs are also being utilized in fields like fitness, entertainment, *etc*. Every WBAN consists of interconnected sensor nodes, which can be deployed on, near or within the human body [1]. These sensor nodes are capable of sensing human vital signs, processing the sensed data and delivering the data to the concerned medical server for treatment. It is of great interest to utilize wireless communication for remote monitoring of a human body. Such monitoring must be unobtrusive, reliable and cost effective, so that the patient can truly benefit from wireless technologies. To achieve these targets, sensors in WBANs must consist of smaller nodes with smaller batteries relative to conventional wireless sensor networks (WSN). Smaller batteries create a restriction on the energy consumed by sensors in sensing, processing, storing and delivering the data, which ultimately affect the overall energy efficiency, throughput and latency of the WBAN. WBANs are required to function accurately and independently for a long period of time without recharging the batteries. Therefore, the design of an energy-efficient and reliable communication protocol, for improving the network lifetime, is one of the major challenges in WBANs. Other factors that may greatly affect data transmission are variable path loss due to shadow or slow fading caused by physical movement of human body parts [2,3]. Therefore, a well-designed network topology in terms of reliable data transmission is also required for WBANs, which may significantly prevent the data loss and wastage of energy.

Cooperative communication is considered to be one of the best solutions to overcome the effects of fading in the wireless channel. In a conventional cooperative communication, a source transmits sensed information to a destination, not only utilizing a direct link, but also utilizing relays/cooperative nodes for transmitting the same data [4]. However, a conventional cooperative communication network makes inefficient use of the channel resources, as relays always forward the source signal to the destination irrespective of the channel conditions [5]. Although the WBAN is usually required to work as a single-hop star network, research has shown improvement in WBANs’ performance by utilizing cooperative relaying schemes. Network throughput may be enhanced by utilizing the broadcast nature of wireless transmission by propagating an independent signal through different paths. The main idea behind this approach is that if a signal experiences noise on a certain path at a particular instant, then other independent paths may carry the same signal with less noise or fading. By introducing the concept of cooperative diversity, both the signal-to-noise Ratio (SNR) and packet error rate (PER) of a signal can be improved at the receiver end. Cooperative links, given a target bit error rate (BER) level, are utilized to improve network lifetime and throughput.

There may be two types of cooperative communication: (i) single relay based and (ii) multiple relay based. In multiple relay-based systems, relay selection may be opportunistic or deterministic according to the needs of the network. In opportunistic selection, a node that forwards a packet is determined on-the-fly and depends on the packet receiving node. Whereas, in the deterministic approach, the node that is supposed to forward the data packet, which is predetermined. Moreover, for a specific relay selection, the relaying strategy can be fixed, selective or incremental. In fixed relaying, relays always forward the received data after processing; whereas selective relaying makes use of instantaneous channel information to decide between relay forwarding and source re-transmission. In incremental relaying, a short feedback, indicating the success or failure of sent data, from the destination is used. Relay(s) is (are) allowed to forward the signal if and only if direct transmission fails; otherwise, the source continues with the transmission of the next data packet. This approach reduces the energy consumption and total transmission time of a network. Incremental relaying protocols are extensions of incremental redundancy protocols, or Hybrid Automatic-Repeat reQuest (HARQ) [5].

In this research, we first analyse three different communication schemes given in [6]. A comparison of direct communication and incremental cooperative communication schemes for both on-body and in-body WBANs is given in this paper. Performance evaluation shows that two-stage incremental cooperative communication performs well in terms of PER at the cost of EE. To further improve the results achieved in [7], we propose three-stage incremental cooperation in WBANs. We derive analytical expressions in terms of PER and EE of the proposed three-stage cooperative communication scheme. Analysis and simulation results for the proposed scheme show that three-stage cooperative communication outperforms two-stage cooperative communication in terms of reliability and PER. Furthermore, we propose a new WBAN protocol, enhanced incremental cooperative critical data transmission in emergencies for static WBANs (EInCo-CEStat), in which we implement three-stage incremental cooperation and compare its performance to existing WBAN protocols: InCo-CEStat [8] and Co-CEStat [9].

Some of the related work and motivation behind this work is discussed in Section 2. Analytical analysis of the proposed scheme in terms of PER and EE is given in Section 3. Section 4 presents the simulation analysis of PER and EE for the proposed scheme. Implementation of these compared and proposed schemes in WBAN protocols is shown in further sections. Section 5 describes the system models of the proposed protocols. Simulation results are shown in Section 6. Finally, conclusion and performance trade-offs are given at the end of the paper.

## 2. Related Work and Motivation

Different cooperative communication schemes are also proposed to make efficient use of available resources. In [8], the authors propose a routing protocol for wildfire monitoring. Cooperative communication is utilized to mitigate the effects of shadowing and to improve network lifetime. Transmission quality is enhanced by sharing network resources between nodes. A technique of reinforced learning by opponent modelling, optimizing a cooperative communication protocol, is used, which is based on the Received Signal Strength Indication (RSSI) and energy consumption of nodes.

In [9], the authors proposed a framework for designing efficient cloud-assisted protocols in ambient assisted living environments. In the proposed framework, two planes are defined for control and data operations. Network coordination tasks are performed on the cloud, whereas data operations are locally held at sensor nodes. The authors designed a Medium Access Protocol (MAC) protocol that utilizes Random Linear Network Coding (RLNC) in a cooperative network environment. The proposed framework is designed for harsh environments with few relays.

The authors in [10] proposed a Hybrid Energy Harvesting polling Media Access Control protocol (HEH-BMAC) for WBANs in which energy is harvested through the human body. The proposed protocol works on priority operation for sensor nodes and provides flexibility for the network. Protocol performance is analysed in terms of energy harvesting rates, packet inter-arrival times and network size. It is observed that HEH-BMAC dynamically adapts its operation according to potential changes in the performance parameters.

The authors in [11] presented a scheme for telemonitoring vital signs, which exploits compressed sensing (CS) for low-complexity signal compression/reconstruction. It utilizes distributed cooperation for reliable data transmission to a body node coordinator. Furthermore, a Cooperative Compressed Sensing (CCS) approach is introduced, which increases the energy efficiency of WBANs by utilizing the benefits of Random Linear Network Coding (RLNC). Energy-efficient RLNC is observed and compared to the store-and-forward (FW) protocol. Mathematical analysis shows that the gain introduced by RLNC increases as the link failure rate increases with a limited number of relays. The authors also proposed a reconstruction algorithm that further enhances the benefits of RLNC by exploiting key characteristics of vital signals. Simulation results show that the proposed recovery algorithm increases the energy efficiency of conventional CS-based reconstruction methods. A cooperative WBAN protocol is proposed in [12], which is able to support multi-hop communication along with cooperation. This protocol extends cooperation at the MAC layer to cross-layered gradient-based routing. WBAN is usually assumed to be a single-hop star network, whereas research shows that conventional multi-hop cooperative relaying has improved the performance of WBANs.

The authors in [13] presented a Cooperative Energy Harvesting MAC protocol (CEH-MAC) that utilizes Energy Harvesting (EH) information to improve the performance of WBANs. The protocol performs according to EH conditions. CEH-MAC allows relay nodes to store sufficient energy to perform cooperation when retransmission is needed. Simulation results show that the proposed scheme outperforms the existing protocol without EH awareness.

In [14], the authors presented a cooperation-based Harvest-Then-Cooperate (HTC) scheme for WSNs. In the proposed scheme, the source and relay harvest energy from the access point in the downlink and utilize cooperation in the uplink to transmit source node data. Throughput performance is investigated in terms of relay number, time allocation and the relays’ positions.

The authors in [15] proposed the Modified Double-Threshold Energy Detection (MDTED) scheme, which utilizes the cooperative sensing spectrum mechanism for WSNs. In this study, location and channel information is considered to improve the clustering mechanism. Simulation results show that the proposed algorithm significantly improves the collaborative sensing ability.

In [16,17], the authors utilize Cooperative Network Coding (CNC) to improve reliability in WBANs. CNC combines cooperative communications and network coding, in a feed-forward architecture. Packets are transmitted over spatially-distinct paths, which significantly improve the network throughput due to extra paths for communication. These proposed schemes also provide enhanced self-healing, which is a required feature in WBANs. Moreover, these feed-forward techniques are mostly suitable for real-time applications, where retransmissions are an inappropriate alternative.

In [18], the authors evaluate the performance of cooperative relaying schemes for improving the robustness of WBANs and take the PER outage probability as the performance parameter. Some sensors are selected to provide redundant links for other nodes having the worst channel conditions. Relay nodes are elected from a statistical perspective.

In [19], the outage performance and Energy Efficiency (EE) of direct transmission and single and multi-relay cooperation schemes are analysed in the context of WBANs. To minimize the energy consumption, the authors study the problem of optimal power allocation with the constraint of the targeted outage probability.

Many other techniques and schemes are implemented for communication in WBANs.

A wireless accelerometer sensor module is used to determine the link performance [20]. It records data and traffic lost on different runners and for different transmitter locations around the human body. The approximate location of nodes is determined for accurate and reliable reception of data. The results also show that the sensor on the wrist gives the best outcome from the locations tested.

In [21], the authors propose a framework for the estimation of network lifetime. A parametric model for a health monitoring network (HMN) is created, and probabilistic analysis is used to determine the timing and the distribution of time failure in the HMN.

In [22], the authors address WBAN data monitoring challenges, allowing virtual groups to be formed between devices and patients, nurses and doctors to enable remote monitoring of WBAN data. A new metric, the quality of health monitoring, is also introduced to provide feedback on the quality of the data received.

In [23], the authors present a Reliability Enhanced-Adaptive Threshold based Thermal-unaware Energy-efficient Multi-hop ProTocol (RE-ATTEMPT) in which positive features of both single-hop and multi-hop communications are utilized. Priority-based routing is implemented in the protocol for normal and critical data transmission. Routes are selected on the basis of the minimum hop count, which reduces the transmission delay.

A reliable anycast routing protocol, for ZigBee-based wireless patient monitoring, is proposed in [24]. Mobile sensor nodes select the closest sink to forward their data in a wireless mesh network (WMN). This protocol reduces the number of control messages with fast re-routing. This scheme also reduces latency by using intermediate routers for route recovery. A device for fall monitoring is also implemented on the basis of the proposed scheme.

In [25], the data transmission scheduling problem is analysed to make use of sleep mode and opportunistic transmission for EE. Propagation channel requirements and delay constrains are considered in the design of the scheduling policy to save the energy of sensor nodes. The Lyapunov optimization formulation is utilized to propose a two-step scheduling algorithm. It is proven that the algorithm can provide worst-case delay, which is guaranteed under certain conditions.

In [26], the authors utilize a low cost wake-up radio module to prolong the network lifetime. This radio module is attached to the sensor node. The lifetime of a network is extended by reducing the power consumption in idle state and increasing the sleep time of sensor nodes. A MAC protocol is proposed for the WBAN, which uses an on-demand wake-up radio through a centralized wake-up mechanism. The results of this proposed method are compared to some of the contemporary MAC protocols.

An efficient technique is presented in [27] to make the operation of battery-powered devices more reliable and efficient with minimal energy consumption. Their scheme combines an efficient antenna design with a cross layer energy-efficient protocol to maximize the network lifetime of WBANs. Towards this goal, an efficient system is designed through which the performance of WBANs is enhanced.

The authors in [28] presented a survey on machine-to-machine (M2M) systems for mHealth applications for wireless communication. The survey focuses on different communication aspects of the M2M architecture. This survey provides a systematic review WBANs and discusses end-to-end solutions involved in the design and implementation of practical mHealth applications.

In [29], the authors addressed the problem of additional delays by relays and decoding failures due to channel errors. The cloud architecture, where the set of relays is connected to a coordinating entity, called the cloud manager, is used to tackle these problems. The paper proposed a Cloud-assisted Linear Network Coding RLNC-based MAC protocol (CLNC-MAC) and develops a mathematical model for the calculation of the key performance metrics: system throughput, mean completion time for data delivery and the energy efficiency. The gain of RLNC is utilized in error-prone channels to show the importance of central coordination.

The authors in [30] presented harvesting energy in the human environment as an effective way to charge the body sensor nodes in WBANs. The authors proposed a joint Power Energy Harvesting-Quality of Service (PEH-QoS) control scheme, which is composed of three modules that interact with each other in order to make optimal use of energy, hence achieving the best possible QoS. The proposed scheme ensures efficient data detection and delivery of medical events by sensor nodes. The simulation results showed that the application of PEH-QoS in a medical node increases the detection efficiency, throughput and the energy efficiency of WBANs.

A survey paper is presented in [31], which focused on the main applications, technologies and standards, problems in WBANs’ design and future aspects. Case studies and simulations are conducted for both real and experimental implementations. The major aim of this survey is to analyse WBANs’ design and to highlight important problems that affect the performance of WBANs.

In [32], the authors presented a relay mechanism with predefined relaying nodes to reduce the possibility of data relaying failure. A predefined relaying node will be active during the data relaying process, even if it is not elected. Simulation results reveal that the proposed relay mechanism is able to achieve higher throughput and network lifetime. The proposed relay mechanism is evaluated in a super-frame structure. Comparison of the state-of-the-art work is also shown in Table 1.

In WBANs, low energy consumption of sensor nodes with reliable and quick delivery of data is of extreme significance. A direct link between transmitter and receiver is appropriate to deliver data from the source to the destination in WBANs. However, links between nodes may experience path-loss due to fading or noise in both Line-Of-Sight (LOS) and Non-LOS (NLOS) scenarios. Low SNR, at any particular time, causes a high packet drop rate. Therefore, an efficient and reliable topology for WBANs is required to ensure the high throughput and low energy consumption of sensor nodes. Conventional cooperative communication proves to be more reliable by providing cooperative links along with a direct link to transmit the same information. To reduce the energy consumed by cooperative nodes in conventional cooperation, the incremental relay-based approach is used to utilize the merits of both direct and cooperative links. This type of cooperative communication increases EE of WBANs. Major objectives behind this research are: (i) to study the effects of incremental relay-based cooperation with a different number of cooperative relays/nodes; (ii) to improve the EE of the conventional cooperative scheme by using the incremental relay-based cooperative scheme; and (iii) to implement incremental cooperation schemes in WBAN protocols to improve the overall network PER and EE.

## 3. Analysis of Three-Stage Incremental Cooperative Communication

**Figure 1:**
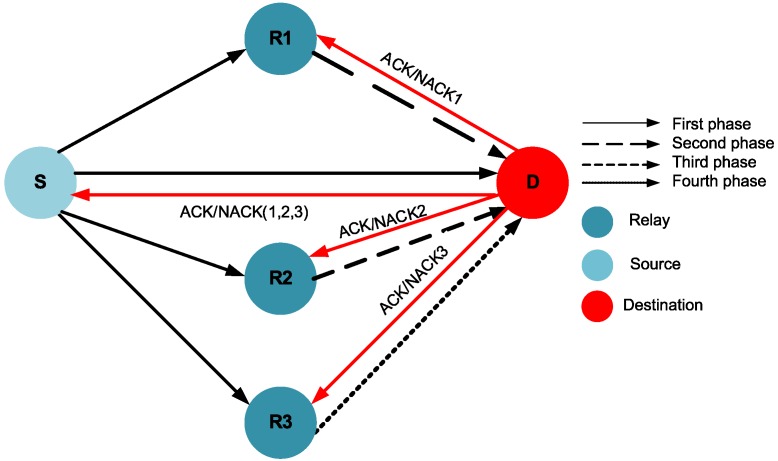
Three-stage incremental cooperative communication.

We consider a WBAN in which the on-body sensor nodes transmit their sensed data to the coordinator/sink attached to the body. As the distances between sensor nodes in WBAN are small, it is assumed that all of the nodes are within the transmission range of each other. Communication is considered to be half-duplex. Figure 1 explains the system model of the proposed scheme.

Our proposed WBAN scheme consists of four communication phases. There are three available potential relays $R_{1}$, $R_{2}$ and $R_{3}$ for a source node. The proposed scheme has a three-stage ARQ mechanism, as shown in Figure 1. In the first phase of cooperation, the source transmits the data packet to the destination, and all three relays overhear this packet. If the destination node successfully decodes the packet in the first phase, it sends a short feedback in the form of positive ACKnowledgement (ACK), which indicates that there is no need for relaying. However, if the destination node fails to correctly decode the data packet, a negative ACKnowledgement (NACK) is sent, which is also heard by all relays. After this, the three-stage relaying process is invoked. If relay $R_{1}$ has correctly received and decoded the data packet in the first phase, it forwards that packet to the destination during the second phase. If the packet is decoded successfully at the destination, it transmits back the ACK (ACK 2), and hence, the first stage of cooperative relaying is successful. Otherwise, the destination node sends the NACK (NACK 2), implying the need for the second stage of cooperative relaying. Upon overhearing NACK 2, relay $R_{2}$ forwards the data packet, which is correctly received in the first phase, to the destination in the third phase. If the destination node is able to decode the packet successfully, it sends back ACK 3; otherwise, it sends NACK 3, which indicates the failure of the second stage of cooperation, as well. It may be noted that even if $R_{1}$ does not transmit in the second phase (due to decoding failure at $R_{1}$), $R_{2}$ can forward the packet to the destination in the third phase, if it had received the packet correctly in the first phase. The same is the case with the third relay $R_{3}$: if $R_{2}$ is unable to decode the packet in the first phase or the destination fails to decode and receive the packet correctly from $R_{2}$, $R_{3}$ is responsible for transmitting that data packet to the destination. If the destination node is able to successfully decode the packet, the success of the third stage of relaying occurs; otherwise, the packet is considered dropped. Figure 1 shows the incremental cooperative communication model for three-stage relaying.

Now, we derive expressions for calculating PER for three-stage incremental relaying. Expressions for single- and two-stage relaying communication schemes may be seen in [6]. We also analyse the overall energy consumption for our proposed cooperative scheme. In WBANs, it is assumed that the link between two nodes is affected by path loss, shadowing and additive white Gaussian noise (AWGN). According to [2], the path loss model for WBANs, which is dependant on the distance *d* between communicating nodes, is based on the Friis formula in free space and is given as:

(1) $$PL\left(d\right)=PL\left(d_{o}\right)+10nlog\frac{d}{d_{o}}$$

where $PL(d_{o})$ is the path loss in dB at a reference distance $d_{o}$ and *n* is the path loss exponent. Path loss due to distance may vary with body movement and certain changes in the surrounding environment. It may differ from its mean value, and this phenomena is called shadowing. Shadowing may also occur in a static body. By considering the factor of shadowing, the total path loss may be given as:

(2) $$PL=PL\left(d\right)+X_{\sigma }$$

Here, $X_{\sigma }$ is a shadowing factor in dB, which is a Gaussian-distributed random variable with zero mean and a standard deviation, *σ* [2]. According to the channel model for WBANs given in [2], the SNR at the receiver end is computed as:

(3) $$\gamma \left(dB\right)=P_{T}-PL-P_{N}$$

where $P_{T}$ is the transmit power and $P_{N}$ is the noise power for all nodes.

### 3.1. PER Analysis

For three-stage incremental relaying, it is assumed that there are three potential relays, $R_{1}$, $R_{2}$ and $R_{3}$, available to cooperate with the source. Let $PER_{SR_{1}}$, $PER_{SR_{2}}$, $PER_{SR_{3}}$, $PER_{R_{1}D}$, $PER_{R_{2}D}$ and $PER_{R_{3}D}$ represent the probability of error of the source-to-relay ($R_{1}$) ($S-R_{1}$), source-to-relay ($R_{2}$) ($S-R_{2}$), source-to-relay ($R_{3}$), ($S-R_{3}$), $R_{1}$-to-destination ($R_{1}-D$), $R_{2}$-to-destination ($R_{2}-D$) and $R_{3}$-to-destination ($R_{3}-D$) links, respectively.

The three-stage relaying process fails if one of conditions mentioned in Table 2 occur.

Hence, the PER for the three-stage relaying scheme is given as:

(4) $$\begin{array}{cc} & PER_{CC}^{\left(3\right)}=PER_{SD}PER_{SR_{1}}PER_{SR_{2}}PER_{SR_{3}}\\ & +PER_{SD}\left(1-PER_{SR_{1}}\right)PER_{SR_{2}}PER_{SR_{3}}PER_{R_{1}D}\\ & +PER_{SD}PER_{SR_{1}}\left(1-PER_{SR_{2}}\right)PER_{SR_{3}}PER_{R_{2}D}\\ & +PER_{SD}PER_{SR_{1}}PER_{SR_{2}}\left(1-PER_{SR_{3}}\right)PER_{R_{3}D}\\ & +PER_{SD}\left(1-PER_{SR_{1}}\right)\left(1-PER_{SR_{2}}\right)\left(1-PER_{SR_{3}}\right)PER_{R_{1}D}\\ & PER_{R_{2}D}PER_{R_{3}D}\\ & +PER_{SD}\left(1-PER_{SR_{1}}\right)\left(1-PER_{SR_{2}}\right)PER_{SR_{3}}PER_{R_{1}D}\\ & PER_{R_{2}D}PER_{R_{3}D}\\ & +PER_{SD}PER_{SR_{1}}\left(1-PER_{SR_{2}}\right)\left(1-PER_{SR_{3}}\right)PER_{R_{1}D}PER_{R_{3}D}\\ & +PER_{SD}\left(1-PER_{SR_{1}}\right)PER_{SR_{2}}\left(1-PER_{SR_{3}}\right)PER_{R_{1}D}PER_{R_{3}D} \end{array}$$

### 3.2. EE Analysis

We analyse the EE for three-stage incremental cooperation according to the energy model given in [14]. This model considers the energy required to run the circuitry of the transmitter and receiver for both data and ACK/NACK packets. The total energy consumed in the transmission of a data packet is computed below for three-stage relaying process.

(5) $$\begin{array}{cc} & EE_{CC,DATA}^{\left(3\right)}=[\left(E_{TX_{elec}}+4E_{RX_{elec}}+\frac{P_{T}}{R}\right)\left(1-PER_{SD}\right)\\ & +\left(2E_{TX_{elec}}+5E_{RX_{elec}}+2\frac{P_{T}}{R}\right)PER_{SD}\left(1-PER_{SR_{1}}\right)\\ & +\left(2E_{TX_{elec}}+5E_{RX_{elec}}+2\frac{P_{T}}{R}\right)PER_{SD}PER_{SR_{1}}\left(1-PER_{SR_{2}}\right)\\ & +\left(2E_{TX_{elec}}+5E_{RX_{elec}}+2\frac{P_{T}}{R}\right)PER_{SD}PER_{SR_{1}}PER_{SR_{2}}\left(1-PER_{SR_{3}}\right)\\ & +\left(2E_{TX_{elec}}+5E_{RX_{elec}}+2\frac{P_{T}}{R}\right)PER_{SD}\left(1-PER_{SR_{1}}\right)PER_{R_{1}D}\\ & PER_{SR_{2}}\left(1-PER_{SR_{3}}\right)\\ & +\left(3E_{TX_{elec}}+6E_{RX_{elec}}+3\frac{P_{T}}{R}\right)PER_{SD}\left(1-PER_{SR_{1}}\right)\\ & PER_{R_{1}D}\left(1-PER_{SR_{2}}\right)PER_{R_{2}D}PER_{SR_{3}}\\ & +\left(4E_{TX_{elec}}+7E_{RX_{elec}}+4\frac{P_{T}}{R}\right)PER_{SD}\left(1-PER_{SR_{1}}\right)\\ & PER_{R_{1}D}\left(1-PER_{SR_{2}}\right)PER_{R_{2}D}\left(1-PER_{SR_{3}}\right)\\ & +\left(E_{TX_{elec}}+4E_{RX_{elec}}+\frac{P_{T}}{R}\right)PER_{SD}PER_{SR_{1}}PER_{SR_{2}}PER_{SR_{3}}]\left(L+H\right), \end{array}$$

where, *L* is the packet size and *H* is the overhead size in bits. $E_{TX_{elec}}$ and $E_{RX_{elec}}$ are the energies required for the transmitter and receiver electronics in transmitting and receiving one bit, respectively. *R* is the data rate.

We find the total energy consumption of all of the events in which packet transmission is successful:

(i) The probability of successful direct transmission is $\left(1-PER_{SD}\right)$. Three relays overhear the packet, which consumes receiving energy, $\left(E_{TX_{elec}}+4E_{RX_{elec}}+\frac{P_{T}}{R}\right)$.
(ii) The direct link S−D fails, and $R_{1}$ correctly receives and decodes the message from the source. $R_{1}$ forwards the packet to the destination with probability $PER_{SD}\left(1-PER_{SR_{1}}\right)$, which results in a total energy consumption per bit of $\left(2E_{TX_{elec}}+5E_{RX_{elec}}+2\frac{P_{T}}{R}\right)$.
(iii) In case S−D and $S-R_{1}$ links fail and the $S-R_{2}$ link is error free. The energy consumption is the same as in (ii).
(iv) In case S−D, $S-R_{1}$ and $S-R_{2}$ links fail and the $S-R_{3}$ link is error free. The energy consumption is the same as in (ii).
(v) The S−D link fails; the $S-R_{1}$ link is error free; and $R_{1}$ decodes and forwards the message to the destination. $S-R_{2}$ and $R_{1}-D$ links fail, and the $S-R_{3}$ link is error free. The probability of this event is $PER_{SD}\left(1-PER_{SR_{1}}\right)PER_{R_{1}D}PER_{SR_{2}}\left(1-PER_{SR_{3}}\right)$, and the energy consumption per bit is the same as in (ii).
(vi) The S−D link fails; $S-R_{1}$ and $S-R_{2}$ links are error free; and $R_{1}-D$, $R_{2}-D$ and $S-R_{3}$ links are in error with a total probability of $PER_{SD}\left(1-PER_{SR_{1}}\right)PER_{R_{1}D}\left(1-PER_{SR_{2}}\right)PER_{R_{2}D}PER_{SR_{3}}$. The energy consumption per bit is $\left(3E_{TX_{elec}}+6E_{RX_{elec}}+3\frac{P_{T}}{R}\right)$.
(vii) The direct link fails; $S-R_{1}$, $S-R_{2}$ and $S-R_{3}$ are error-free links; whereas, $R_{1}-D$ and $R_{2}-D$ links are in error with a total probability of $PER_{SD}\left(1-PER_{SR_{1}}\right)PER_{R_{1}D}\left(1-PER_{SR_{2}}\right)PER_{R_{2}D}\left(1-PER_{SR_{3}}\right)$. The energy consumption per bit is $\left(4E_{TX_{elec}}+7E_{RX_{elec}}+4\frac{P_{T}}{R}\right)$.
(viii) All four links from the source to the destination and relays fail with probability $PER_{SD}PER_{SR_{1}}PER_{SR_{2}}PER_{SR_{3}}$. The energy consumption per bit for this event is $\left(E_{TX_{elec}}+4E_{RX_{elec}}+\frac{P_{T}}{R}\right)$.

Total energy consumption also includes the energy involved in the transmission of ACK/NACK packets and is computed as follows:

(6) $$\begin{array}{cc} & EE_{CC,ACK/NACK}^{\left(3\right)}=[\left(E_{TX_{elec}}+4E_{RX_{elec}}+\frac{P_{T}}{R}\right)\\ & +\left(E_{TX_{elec}}+4E_{RX_{elec}}+\frac{P_{T}}{R}\right)PER_{SD}\left(1-PER_{SR_{1}}\right)\\ & +\left(E_{TX_{elec}}+3E_{RX_{elec}}+\frac{P_{T}}{R}\right)PER_{SD}\left(1-PER_{SR_{1}}\right)\left(1-PER_{SR_{2}}\right)PER_{R_{1}D}\\ & +\left(E_{TX_{elec}}+3E_{RX_{elec}}+\frac{P_{T}}{R}\right)PER_{SD}PER_{SR_{1}}\left(1-PER_{SR_{2}}\right)\\ & +\left(E_{TX_{elec}}+3E_{RX_{elec}}+\frac{P_{T}}{R}\right)PER_{SD}PER_{SR_{1}}\left(PER_{SR_{2}}\right)\left(1-PER_{SR_{3}}\right)\\ & +\left(E_{TX_{elec}}+3E_{RX_{elec}}+\frac{P_{T}}{R}\right)PER_{SD}\left(1-PER_{SR_{2}}\right)PER_{SR_{1}}PER_{R_{2}D}\left(1-PER_{SR_{3}}\right)]\left(A+H\right) \end{array}$$

where *A* is the size of ACK/NACK in bits. Energy consumption is the same for the transmission and reception of both ACK and NACK. The first term in Equation (6) shows the energy consumption involved in the transmission of ACK/NACK by the destination in the first phase. (1 − $PER_{SD}$) is the probability of ACK and NACK being transmitted with probability $PER_{SD}$. In the second phase, either ACK or NACK is transmitted for the the packet decoded and forwarded by $R_{1}$ to the destination; this happens with probability $PER_{SD}$ (1 − $PER_{SR_{1}}$). The second term in Equation (6) represents the energy consumption associated with the second phase. In the third phase, $R_{2}$ forwards the packet, which is followed by another sequence of ACK/NACK transmissions. ACK/NACK is transmitted if $R_{2}$ decodes and forwards the packet to the destination. This may happen due to the following reasons: (i) failure of direct communication, one-stage relaying, the $S-R_{2}$ link becoming error free; and (ii) failure of S−D and $S-R_{1}$ links, the $S-R_{2}$ link becoming error free. In the fourth and last phase, ACK/NACK is transmitted if $R_{3}$ decodes and forwards the packet to the destination. This may happen due to the following reasons: (i) failure of S−D, $S-R_{1}$ and $S-R_{2}$, the $S-R_{3}$ link is error free; and (ii) failure of S−D and $S-R_{1}$, the success of $S-R_{2}$, the failure of the $R_{2}-D$ link, and the $S-R_{3}$ link is error free. Therefore, the EE of the three-stage incremental cooperative communication is computed as follows:

(7) $$\begin{array}{cc} \eta_{CC}^{\left(3\right)} & =\frac{(1-PER_{CC}^{3})xL}{E_{CC}^{3}+EE_{CC,ACK/NACK}^{\left(3\right)}} \end{array}$$

where *x*=$E_{TX_{elec}}$ + $4E_{RX_{elec}}$ + $\frac{P_{T}}{R}$.

## 4. Simulation Analysis of PER and EE for Three-Stage Incremental Cooperative Communication

In this section, we present the performance evaluation of our proposed work, which is compared to the existing schemes in [6], in terms of PER and EE. All results are obtained from the expressions presented in Section 6. Simulation parameters are given in Table 3 and Table 4. We only consider the case of on-body sensor nodes, as we further implement these models in on-body WBAN protocols.

### 4.1. PER

**Figure 2:**
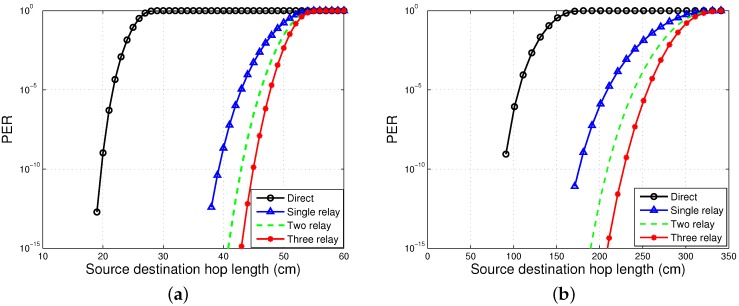
Packet error rate (PER) analysis. (**a**) PER for on-body NLOS communication; (**b**) PER for on-body LOS communication.

PER is plotted against distance to see the effect of various distances between the source and destination, $r_{sd}$. For cooperative communication, the distances between the source and relay, $r_{sr}$, and the relay and destination, $r_{rd}$, are kept half of the distance between the source and destination. Figure 2a,b shows the PER for on-body LOS and NLOS, the direct and cooperative communication schemes. It is observed from Figure 2 that PER for direct communication is higher than the PER for cooperative communication. When the direct link is not reliable enough for efficient transmission, cooperative communication proves to be a better solution by providing redundant links for packet transmission. It is seen from the figures that path loss increases with the increase in distance. Therefore, for a larger distance, direct communication has more PER. Thus, for the increased hop length between the source and destination, cooperative communication is useful. It is obvious from the plots that two-relay communication is better than single-relay communication; the same is the case for three-relay communication, which is better than single- and double-relay communication. When the first and second stage of relaying fails, relay $R_{3}$ provides an extra redundant link to the destination and enhances network reliability. It is also shown that the PER of LOS communication is less than NLOS communication due to more path loss in NLOS communication. Therefore, LOS communication offers less PER for longer hop lengths between the source and destination.

### 4.2. EE

**Figure 3:**
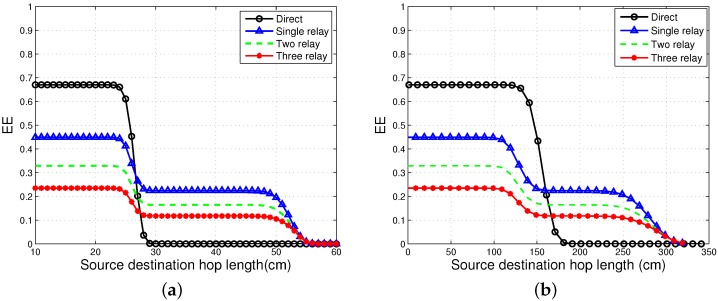
Energy efficiency (EE) analysis. (**a**) EE for on-body NLOS communication; (**b**) EE for on-body LOS communication.

The EE of direct and the incremental cooperative communication schemes is observed in Figure 3a,b. A series of experiments are performed in [6] to find the best distance between the source and relay and between the relay and destination. The hop lengths of S−R and R−D links are selected to be 0.5-times the distance between the source and the destination. Figure 3 shows the results for the EE of on-body NLOS and LOS scenarios, respectively. EE is plotted against S−D hop length, $r_{sd}$, for the direct and cooperative communication schemes.

It is concluded from the results that increased distance between the source and destination, $r_{sd}$, decreases EE. For lower distances, direct transmission proves to be considerably more energy efficient than cooperative transmissions. When the hop length, $r_{sd}$, exceeds a certain threshold, cooperative communication turns out to be more energy efficient than direct communication. Although cooperative communication improves reliability, as it has lower PER, its EE is significantly affected, because of the increased energy consumption due to additional transmissions and decoding by the relays. When $r_{sd}$ is above the threshold, the PER of direct communication is so high that its EE is significantly affected. As the PER of three-relay communication is lower than the other two relaying schemes, it has the lowest EE due to more energy consumption by the relays.

## 5. Incremental Cooperative Routing Protocols for WBANs

To study the effects of three-stage incremental cooperation, we design the EInCo-CEStat protocol and compare its results to InCo-CEStat [7] and Co-CEStat [8]. The conventional cooperation is used in Co-CEStat in which the same data are forwarded by both relays. Whereas, in InCo-CEStat, incremental cooperation is used in which data are forwarded incrementally by relay nodes. However, both schemes in [7,8] utilize two relays for a single source node. EE, PER, throughput and the stability period for all three protocols are observed. We consider four-phase incremental relay-based cooperation in EInCo-CEStat by using three potential relays for each source node. In the first phase, the source transmits data to the sink, which is overheard by its three potential relays, $R_{1}$, $R_{2}$ and $R_{3}$. If the destination/sink is able to detect the packet correctly in this phase, it sends back an ACK, and the relays just remain idle. If the NACK is received from the sink at the source node, it indicates that the data packet is dropped due to high BER, and data forwarding from $R_{1}$ is needed. If $R_{1}$ successfully detects the data packet in the first phase, it forwards the data packet to the destination (sink) in the second phase. If the data packet is received with acceptable BER at the sink, the second phase of cooperation is successfully completed. However, if the sink fails to detect the packet sent by $R_{1}$, $R_{2}$ is supposed to forward the packet to the sink, which was correctly received in the first phase. If the sink again fails to receive the packet from $R_{2}$ due to high BER, failure of the third phase occurs. Finally, the last phase of communication occurs between $R_{3}$ and sink. The system model for InCo-CEStat is also the same with two-relay cooperative communication. Therefore, InCo-CEStat consists of three communication phases accordingly.

**Figure 4:**
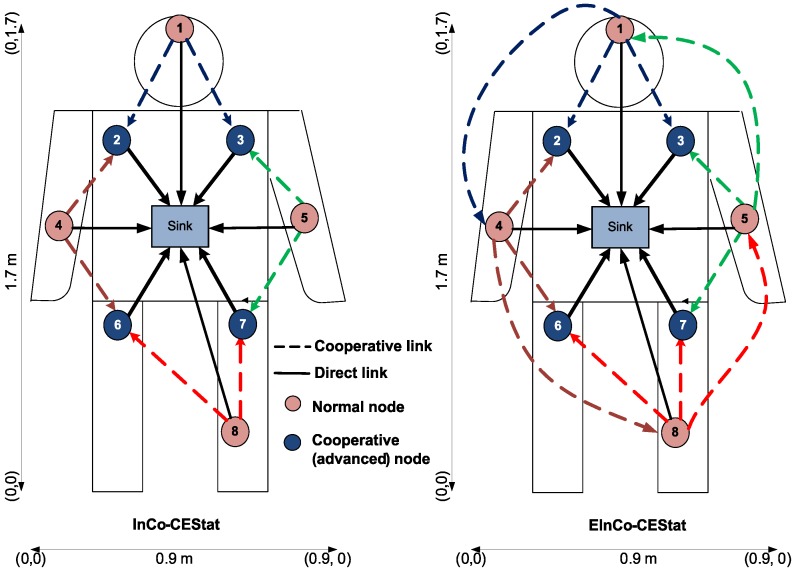
Network topology of incremental cooperative critical data transmission in emergencies for static WBANs (InCo-CEStat) and enhanced InCo-CEStat (EInCo-CEStat).

**Figure 5:**
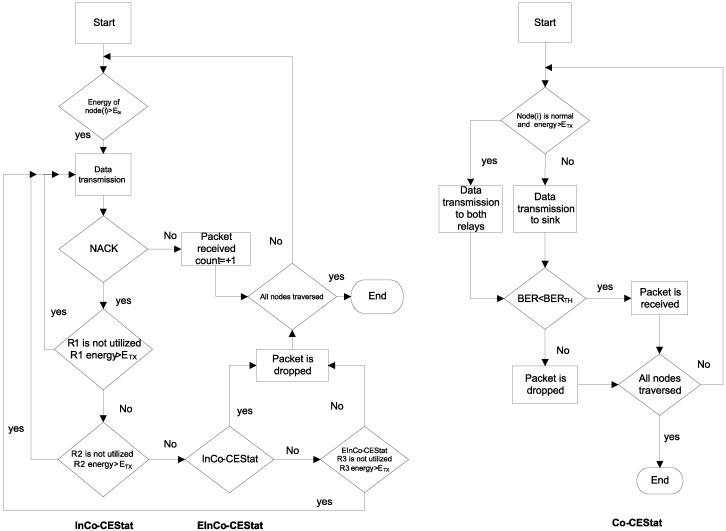
Communication flow diagram of InCo-CEStat, EInCo-CEStat and Co-CEStat.

Topologies of InCo-CEStat and EInCo-CEStat protocols are shown in Figure 4 for comparison. As Co-CEStat has the same topology as InCo-CEStat, it is not mentioned in the figure. Communication flow diagram is also shown in Figure 5 for the compared protocols. There are four normal source nodes (S), four cooperative nodes (R) and a sink node (D) in the proposed and compared WBAN. Cooperative nodes, which are responsible for relaying the data of other nodes, are equipped with higher energy than normal source nodes. As cooperative nodes also have their own sensed data to be transmitted along with data to be forwarded, the selection of the relays is deterministic, *i.e.*, each source node has its own pre-defined relay node for forwarding its data to the sink. Predefined/deterministic relay selection has been given priority over random/opportunistic relay selection due to two reasons: (i) to avoid imbalanced energy consumption of the nodes; and (ii) to minimize end-to-end delay, as WBAN data are critical.

Some assumptions are considered for the simulations as follows [6,9,10]:

1. The sink limits all nodes to transmit only in their own reserved time slots: if all nodes transmit simultaneously to the sink, a data collision may occur, which ultimately causes the loss of data and energy wastage. Thus, collision avoidance and network coordination are not only important for efficient energy consumption, but also to maintain QoS, as sensed data are always critical in WBANs.
2. Half-duplex communication is considered: since the sink does not send data back towards the source nodes, the consideration of half-duplex communication leads to enhanced management of resources; especially energy efficiency.
3. All nodes are within the transmission range of each other: we have considered a human body of dimensions 0.9 m × 1.7 m. Thus, all nodes lie within the transmission range of each other.
4. The time division multiple access (TDMA) scheme is utilized, and the channel is accessed by nodes in different time slots: being energy efficient, TDMA as a channel access scheme is utilized by nodes in different time slots to avoid data collision.

## 6. Simulation Results and Discussion

We compare the performance of the incremental relay-based cooperation implemented in InCo-CEStat and EInCo-CEStat, with an existing cooperative protocol, Co-CEStat. We assume a network area of 0.9 m × 1.7 m, where on body nodes are deployed at fixed positions, as shown in Table 5, and the sink is placed at the centre of the body, *i.e.*, (0.4 m, 0.8 m). Co-CEStat and InCo-CEStat use two relays for cooperative communication, whereas EInCo-CEStat uses three relays for the same purpose. Table 6 shows the simulation parameters considered for all three schemes.

The rest of the simulation parameters are the same as given in Table 3 and Table 4.

### 6.1. Network Performance Parameters: Definitions

- Stability period: In WBANs, the stability period is usually defined as a time interval between the start of a network and the time at which the first node dies.
- Residual energy: The average total remaining energy per second of a network is called the residual energy of the network.
- Network lifetime: The total time duration of a network operation, from the network establishment till the death of the last node, is called the network lifetime.
- Throughput: The total number of successfully-received packets per unit time at the sink is called the throughput.

### 6.2. Network Performance Parameters: Discussions

In this section, we discuss the performance parameters for our proposed protocol in comparison to our selected existing protocol.

#### 6.2.1. Stability Period and Network Lifetime

**Figure 6:**
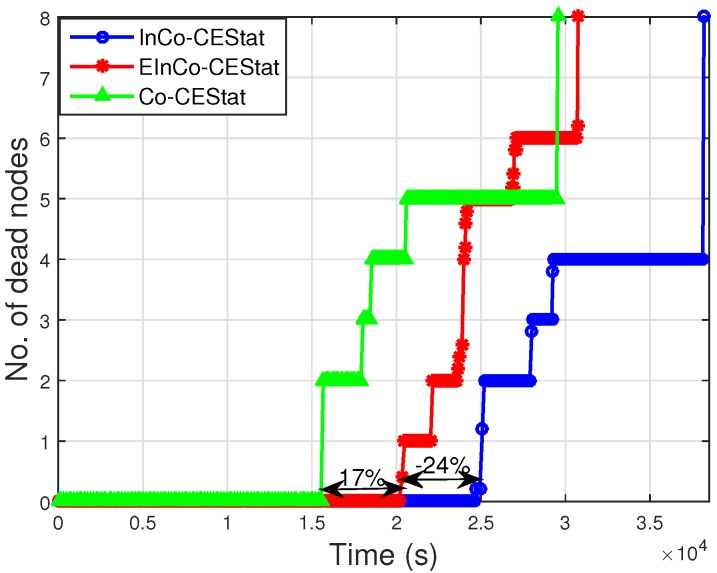
Stability period and network lifetime.

Figure 6 shows the stability period and network lifetime of the compared protocols. It is observed from the figure that the existing cooperative scheme, Co-CEStat, has less of a stability period than InCo-CEStat and EInCo-CEStat. In Co-CEStat, cooperative nodes always forward the data irrespective of the channel conditions. In this strategy, nodes consume more energy in extra transmissions of the same data. Each cooperative node forwards the data of two other nodes along with its own data.

The burden on cooperative nodes reduces as the normal source node dies, thereby making the network stable from $2\times 10^{4}$ s to $2.9\times 10^{4}$ s in InCo-CEStat. It is obvious from Figure 6 that incremental relay-based approaches prove to be more stable with greater network lifetime. The reason is that incremental relaying adapts to the channel conditions, and relays forward the data only when they is needed. EInCo-CEStat has 24% less of a stability period than InCoCEStat. This is due to the extra energy consumption by the third node, which acts as a relay when two stages of cooperative relaying are unsuccessful. The third relay node, which is invoked for forwarding the data in the fourth phase, is always a normal source node having less residual energy than cooperative nodes. Therefore, the first node in EInCo-CEStat dies earlier than in InCo-CEStat, which has only two advanced nodes as cooperative nodes. In InCo-CEStat and Co-CEStat, some nodes remain alive for a greater duration of time due to the extra energy assigned to them at the start of the network. After the death of normal source nodes, the burden on cooperative/advanced nodes reduces, which makes the network stable for a certain period.

#### 6.2.2. Throughput and Packet Drop Rate

**Figure 7:**
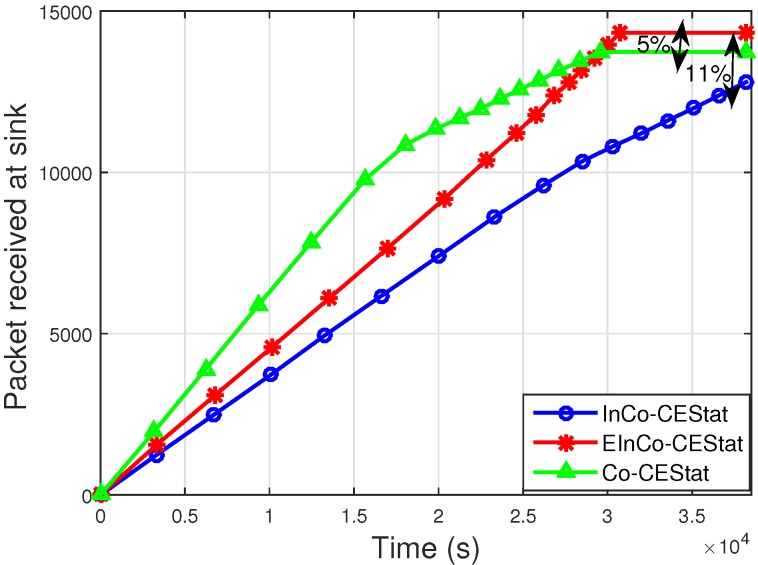
Number of packets received successfully at the sink.

**Figure 8:**
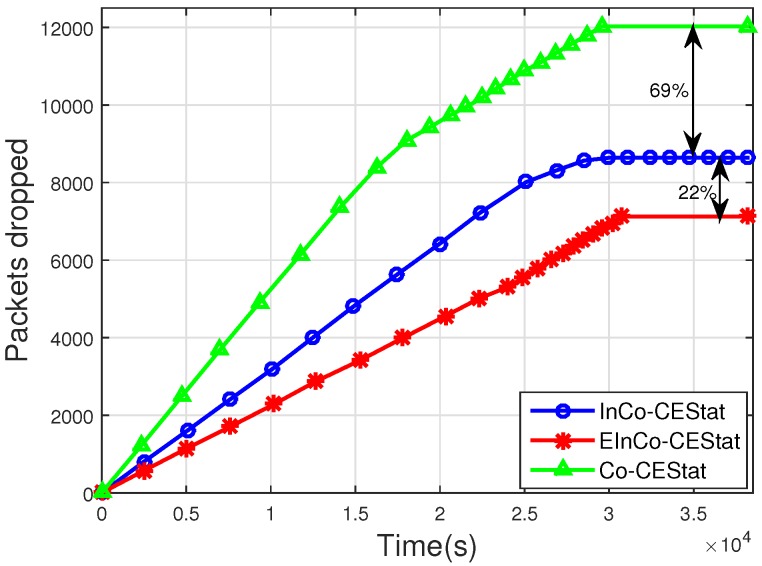
Number of packets dropped.

Figure 7 and Figure 8 show the results for the total number of packets successfully received at the sink and the number of packets dropped due to a higher BER than a certain pre-defined threshold, respectively. It is observed from Figure 7 that EInCo-CEStat has greater throughput than InCo-CEStat. This is due to the availability of more links for packet transmission, in the case of the failure of the direct link. InCo-CEStat has two cooperative nodes, whereas EInCo-CEStat has three cooperative nodes to forward the data of normal nodes. Therefore, higher throughput is achieved by increasing the diversity order in EInCo-CEStat at the cost of EE. Co-CEStat has the highest throughput due to the reception of two copies of transmitted data at the sink. In protocol; Co-CEStat, cooperative nodes always forward the data, so in the ideal scenario, the sink receives multiple copies of the original data sent by the normal nodes.

The packet drop rate of EInCo-CEStat is also less than that of InCo-CEStat, as shown in Figure 8. When direct communication between the source and sink nodes fails, the other three links are available to forward the data to the sink; whereas, InCo-CEStat completes its relaying in three phases and has two more redundant links after the failure of the direct link. The simulation results shown in Figure 2a and Figure 3a reveal this fact. The packet drop rate of Co-CEStat is also greater than the incremental cooperative communication protocols. A greater number of transmissions leads to a greater number of link failures and consequently more dropped packets.

#### 6.2.3. Residual Energy of the Network

**Figure 9:**
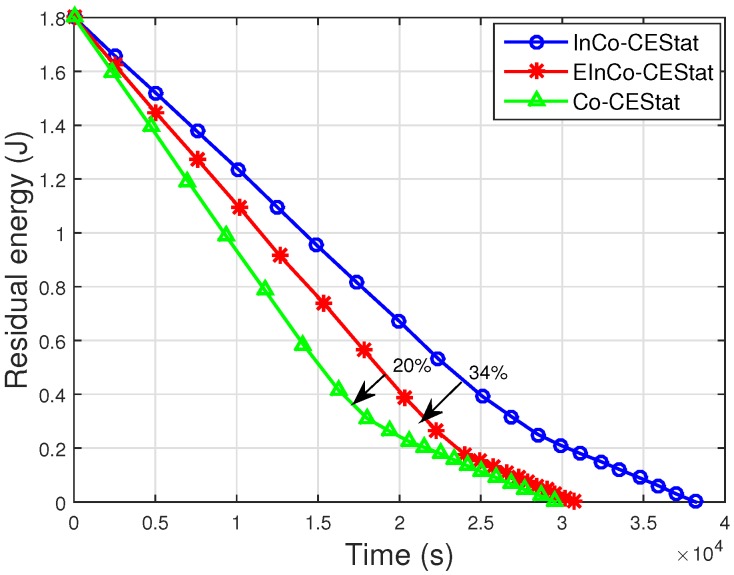
Residual energy of the network.

The residual energy of the network by all the three schemes being analysed is shown in Figure 9. The EE of Co-CEStat is improved by using incremental cooperation in InCo-CEStat and EInCo-CEStat. It is already shown in Figure 2 that two-relay incremental cooperation is more energy efficient than three-relay incremental cooperation. Figure 9 supports the results in Figure 3. In order to reduce PER and to achieve a higher diversity order, three cooperative links are used in EInCo-CEStat, which consumes more transmission and reception energy than InCo-CEStat with two cooperative links. However, EInCo-CEStat is still more energy efficient than Co-CEStat. Therefore, EInCo-CEStat and Co-CEStat have increased throughput at the cost of increased energy consumption. In Co-CEStat, cooperative nodes always forward the data and consume energy, even when it is not needed. Therefore, it is seen from Figure 9 that at any instant of time, InCo-CEStat has the highest residual energy, which leads to the highest network lifetime. Incremental relaying saves the channel resources; however, extra energy is consumed in redundant transmissions.

### 6.3. Performance Trade-Offs Made by the Routing Protocols Being Analyzed

In this section, we discuss the achievements of the proposed protocols and mention the price at which these achievements are made. The proposed protocols are compared to the already designed protocols Co-CEStat and InCo-CEStat. Performance tradeoffs between compared protocols are also shown in Table 7. As discussed earlier, Co-CEStat utilizes the normal cooperation approach in which data are sent to the destination through all of the possible relaying paths regardless of the channel conditions. Normal source nodes send sensed data to the sink through two advanced nodes. In this way, the burden on advanced nodes increases as they always have to send forwarded data along with their own data. Co-CEStat achieves high throughput due to the possibility of the reception of multiple copies of the same data at the sink. Along with the direct link, the availability of two more paths increases the number of received packets at the sink. However, Co-CEStat consumes extra energy of the overall network by transmitting multiple copies of the data through different paths without any need. Therefore, the high throughput of Co-CEStat is achieved at the cost of high energy consumption, as shown in Figure 6 and Figure 9.

To reduce the energy consumption of Co-CEStat, the incremental cooperation approach is utilized in InCo-CEStat and EInCo-CEStat. Incremental relaying in InCo-CEStat exploits a short feedback message from the destination to the source node. The cooperation among nodes process is adaptable to channel conditions. In this type of cooperation, nodes broadcast data to the destination and relay nodes. Relays are responsible for forwarding that data only when the destination sends a NACK to the source node, informing of the failure of the reception of the data packet. Two relay nodes are available for each normal node. The advantage of incremental cooperation is that the second relay forwards the stored data only when the first relay fails to transmit the data successfully. This strategy considerably reduces the overall energy of the network by reducing the extra energy consumption of relay nodes in needless relaying. This reduction in energy consumption is achieved at the cost of a greater number of retransmissions and extra processing in utilizing the short feedback message in the form of ACK/NACK. It is shown in Figure 9 that at some particular time, Co-CEStat has a greater number of packets than InCo-CEStat and EInCo-CEStat.

In the case when the direct link fails, InCo-CEStat utilizes two more retransmissions; whereas, EInCo-CEStat utilizes three more retransmissions when the direct link between the source and destination nodes fails. EInCo-CEStat also utilizes a third relaying path for the normal nodes’ data transmission. This path is only utilized if the first two relays fail to transmit data successfully. Therefore, EInCo-CEStat achieves higher throughput than InCo-CEStat at the cost of the extra energy consumption of the third relay, which can be seen in the simulation results. The low PER of EInCo-CEStat is achieved at the cost of low energy efficiency. This is analysed through the simulation plots. However, InCo-CEStat achieves a greater stability period than EInCo-CEStat due to the lesser energy consumption in the relaying process than that of EInCo-CEStat and Co-CEStat, as shown in Figure 8.

## 7. Conclusions

In this study, we have analysed incremental cooperative communication for WBANs. Incremental cooperation for a single pair of the source and destination, with different numbers of cooperative relay nodes, is analytically studied. We proposed a three-stage incremental relaying scheme with three relay nodes. Analytical expressions are derived for the three-stage incremental relaying scheme and compared to the single- and two-stage relaying schemes. Simulation results show that the proposed three-stage relaying scheme gives high throughput with reduced PER in the presence of three redundant links for a single source node.

Later on, the proposed three-stage incremental cooperation scheme is implemented in the network layer protocol designed for WBANs. Its performance is compared to the conventional cooperative protocol, Co-CEStat, and the incremental cooperation protocol, InCo-CEStat. Simulation results reveal that the less throughput and high packet error rate of Co-CEStat is improved in EIn-CoCEStat by utilizing three-stage incremental relaying. Co-CEStat has a data redundancy problem at the sink due to conventional cooperation, which ultimately causes high energy consumption. Data redundancy at the sink is avoided by using incremental cooperation, so that relays forward the data only whenever it is needed. Hence, need-based incremental relaying helps to improve the data redundancy and data traffic load. The energy consumption of Co-CEStat is also high because of redundant data transmissions. Therefore, by eliminating data redundancy, energy efficiency is increased by EIn-CoCEStat. Furthermore, InCo-CEStat has less throughput and high PER, which are improved by EIn-CoCEStat by utilizing three-stage incremental relaying. Hence, analytical analysis and simulation results positively correlate with each other.

## Figures and Tables

**Table 1:** Comparison of the state-of-the-art work. RLNC, Random Linear Network Coding; CEH, cooperative energy harvesting; HTC, harvest-then-cooperate; MDTED, modified double-threshold energy detection.

Technique	Feature	Domain	Flaws/Deficiencies	Results Achieved
Received Signal Strength Indication (RSSI) [8]	Cooperative routing, Consideration of QoS and energy consumption, Use of Motivated Reinforcement Learning (MRL) algorithm	WSNs, Wildfire monitoring, Shadowing effect of trees	Greater percentage of delayed packets, More average delay to sink, Restricted to a single sink	Better energy consumption, More Network lifetime
Cloud-assisted Random Network Coding (CRNC-MAC) [9]	Utilization of cloud computing to enhance the performance of the cooperative scheme based on network coding	WBANs, Cloud assisted	Latency in cloud communication and bad channel conditions decrease throughput	Enhanced throughput and energy efficiency in error-prone channels
Human Energy Harvesting (HEH-MAC) [10]	Human energy harvesting protocol, polling and probabilistic contention	WBANs, Hybrid polling MAC protocol	No comparison with other MAC protocols, no analytical performance evaluation	improved energy efficiency and delay
Random Linear Network Coded-Aided Cooperative Compressed Sensing (RLNC-ACCS) [11]	compressed sensing and distributed cooperation for reliable data transmission	WBANs, cooperative compressed sensing	Less throughput in error-prone channels	Increased energy efficiency of sensor nodes
CEH-MAC [13]	Exploits energy harvesting information for communication	WBANs, Cooperative MAC scheme that exploits energy harvesting information	No network channel coding and analytical performance evaluation	Improved energy efficiency and network throughput
HTC [15]	Scheme for High SNR radio, Energy-harvesting	WSNs, Cooperation-based networks	SNRs of the source-AP link and all source-relay-AP links mutually correlated, essentially different from conventional cooperative networks with independent link SNRs	Impacts of time allocation, relay number and relay position, on the throughput

MDTED [16]	Cooperative Spectrum Sensing Scheme, Location and Channel-information dependent	Cognitive WSNs, Cooperation-based networks	Based on a single authorization user, number of nodes are fixed, and the value still needs to be computed	Detection Accuracy, Improved collaborative sensing ability
Cooperative routing [20]	Optimal power allocation according to posture information	WBANs	no consideration for end-to-end delay	Improved energy efficiency
Probabilistic analysis [23]	Parametric model for health monitoring with probabilistic approach	WBANs	No realistic scenario, no consideration for end-to-end delay	Improved network lifetime
RE-ATTEMPT [23]	Direct and multi-hop Communication	WBANs	No retransmission of failed data packets, low throughput	Energy Efficient and greater network lifetime
ZigBee-Based [24]	Zigbee device for fall monitoring, utilizes anycast routing	WBANs	High energy consumption	Low transmission latency and control overhead, reliable data delivery
Power-efficient MAC protocol [26]	wake-up table for normal communication, on demand external wake-up radio for emergency	WBANs	No QoS analysis, no multi-hop communication	Efficient in terms of power consumption and delay
CLNC-MAC [29]	Cloud-based coordination by using the RLNC technique	WBANS, cloud-assisted scheme	Increased end-to-end delay, increased complexity	collision avoidance, reliable data delivery with energy efficiency
PEH-QoS [30]	QoS-aware energy management, only a useful data sequence is transmitted	WBANs, Human energy harvesting WBAN	Higher energy consumption in ECG detection	Improved throughput, detection efficiency, end-to-end delay
Opportunistic relay protocol [32]	Predefined relaying nodes for data transmission	WBANs	Delayed transmission and extra energy consumption in relaying	Improved packet delivery rate

**Table 2:** Conditions for the failure of the three-stage relaying process.

Error-Free Links	Failed Links	Remarks
No link	S−D, $S-R_{1}$, $S-R_{2}$ and $S-R_{3}$	No communication; the packet is dropped
$S-R_{1}$	S−D, $S-R_{2}$ and $S-R_{3}$	$R_{1}$ decodes and forwards, but $R_{1}-D$ link fails
$S-R_{2}$	S−D, $S-R_{1}$ and $S-R_{3}$	$R_{2}$ decodes and forwards the data packet, but $R_{2}-D$ link fails
S−D, $S-R_{1}$, $S-R_{2}$ and $S-R_{3}$	S−D, $S-R_{1}$, $S-R_{2}$ and $S-R_{3}$	No more available link
$S-R_{1}$ and $S-R_{2}$	S−D and $S-R_{3}$	$R_{1}-D$ and $S-R_{2}$ links fail
$S-R_{2}$ and $S-R_{3}$	S−D and $S-R_{1}$	$R_{2}$ and $R_{3}$ decode and forward the packet, but $R_{2}-D$ and $R_{3}-D$ link fail
$S-R_{1}$ and$S-R_{3}$	S−D and $S-R_{2}$	$R_{1}$ and $R_{3}$ decode and forward the packet, but $R_{1}-D$ and $R_{3}-D$ links fail

**Table 3:** Simulation parameters. NACK, non-ACK.

Parameter	Value
Packet size	500 bits
Overhead	80 bits
ACK/NACK	64 bits
Transmission power	−12 dBm
Data rate	2 Mbps
$E_{TX_{elec}}$	50 nJ/bit
$E_{RX_{elec}}$	50 nJ/bit

**Table 4:** Channel model parameters. NLOS, non-LOS.

Parameters	NLOS	LOS
$d_{o}$ (cm)	10	10
PL($d_{o}$)(dB)	48.4	35.2
n	5.9	3.11
$X_{\sigma }$	5	6.1

**Table 5:** The coordinates of nodes deployed on the human body.

Node No.	X-Axis (m)	Y-Axis (m)
1	0.45	1.6
2	0.2	1.5
3	0.7	1.5
4	0.1	0.85
5	0.8	0.85
6	0.2	0.5
7	0.7	0.5
8	0.7	0.3

**Table 6:** Simulation parameters for WBAN protocols.

Parameter	Value
Number of nodes	8
Number of sink	1
Initial energy	Cooperative node: 0.3 J
	Normal node: 0.15 J
Offered load	10,000 bits/node
Average wait time [33]	4 s/packet
BER threshold	0.5

**Table 7:** Performance trade-offs made by the routing protocols.

Protocol	Routing Technique	Advances Achieved	Price to Pay
Co-CEStat	Cooperation with two relays	High throughput. (Figure 7)	Decreased stability period and network lifetime. (Figure 6) Low energyefficiency.
InCo-CEStat	Incremental cooperation with two relays	High energy efficiency. (Figure 9) More throughput than Co-CEStat. (Figure 7)	More PER than EInCo-CEStat. (Figure 8)
EInCo-CEStat	Incremental cooperation with three relays	Higher throughput than InCo-CEStat.(Figure 7) Less PER.	Decreased stability period. (Figure 6) Low energy efficiency. (Figure 9)
